# Data analytics competency and religiosity influence on external auditors’ performance in Malaysia

**DOI:** 10.12688/f1000research.73206.1

**Published:** 2021-11-09

**Authors:** Nahariah Jaffar, Abdul Aziz Bin Ahmad, Noor Adwa Sulaiman

**Affiliations:** 1 Faculty of Management, Multimedia University, Persiaran Multimedia, Cyberjaya, Selangor, 63100, Malaysia; 2 Faculty of Business and Accountancy, University of Malaya, Kuala Lumpur, 50603, Malaysia

**Keywords:** Data analytics, competency, religiosity, external auditors, performance, Malaysia, Muslim, non-Muslim

## Abstract

**Background - **Data analytics can support the external auditors’ judgements. However, little is known about the external auditors’ data analytics competency. Likewise, role of religiosity in enhancing the external auditors’ performance is also inadequately investigated. This study examined: 1) the effects of data analytics competency on the external auditors’ performance, and 2) the moderating effects of religiosity on data analytics competency and external auditors’ performance relationship.

**Methods – **Survey was conducted on 201 external auditors. Data analytics competency dimensions, namely, personal capabilities, professional expertise, technical skills, technologies and tools expertise were examined. Religiosity was measured by level and dimension (faith, virtue and optional).

**Results** – Data analytics competency (personal capabilities) has a positive significant effect on the Muslim external auditors’ performance. However, data analytics competency does not affect the performance of non-Muslim external auditors. Level of religiosity has significant moderating effect on the relationship between data analytics competency (technologies and tools expertise) and Muslim external auditors’ performance. Nonetheless, level of religiosity does not moderate the relationship between data analytics competency and the performance of non-Muslim external auditors. Religiosity (virtue) has significant moderating effect on the relationship between data analytics competency (personal capabilities) and Muslim external auditors’ performance. Meanwhile, religiosity (faith) has significant moderating effect on the relationship between data analytics competency (technologies and tools expertise) and non-Muslim external auditors’ performance.

**Conclusion** – This study demonstrates that data analytics competency and religiosity can influence the external auditors’ performance.

## Introduction

In this Fourth Industrial Revolution era, external auditors (EAs) are expected to acquire data analytics competency (DAC) because it can strengthen audit quality (e.g. ICAEW, 2016; Goh, 2017). However, evidence regarding the DAC of EAs and its impact on the external auditors’ performance (EAP) is limited ( Yeo & Carter, 2017). Furthermore, literature suggests religiosity influences individual’s behaviour which includes commitment and ethics (e.g. Van Buren, Syed & Mir, 2020). Nonetheless, little is known about the role of religiosity in enhancing the EAP in the context of DAC. Thus, this study aims: (1) to investigate the influence of DAC on the EAP, and (2) to examine the moderating effect of religiosity on the relationship between DAC and the EAP.

## Competence performance theory and hypotheses

This study adopts Competence Performance Theory (CPT) by Korossy (1999). CPT establishes a connection between competency and performance level. In this study, perceived DAC (personal capabilities, professional expertise, technical skills, and technologies and tools expertise) aids to predict the EAP. Low in certain DAC would affect EAP. Thus, this study hypothesises:

H1a: DAC (personal capabilities) has positive effects on the Muslim/non-Muslim-EAP.

H1b: DAC (professional expertise) has positive effects on the Muslim/non-Muslim-EAP.

H1c: DAC (technical skills) has positive effects on the Muslim/non-Muslim-EAP.

H1d: DAC (technologies and tools expertise) has positive effects on the Muslim/non-Muslim-EAP.

Ali, Baluch and Mohamed Udin (2015) proposed a moderating role of religiosity on the relationship between technology readiness and diffusion of electronic commerce. This suggests that religiosity may influence the consistency of behaviour exhibited by the EAs. It is predicted that high DAC and level of religiosity will enhance the EAP. Therefore, this study further hypothesizes:

H2a-b: Religiosity (level/dimension) moderates the relationship between DAC (personal capabilities) and the Muslim/non-Muslim-EAP.

H3a-b: Religiosity (level/dimension) moderates the relationship between DAC (professional expertise) and the Muslim/non-Muslim-EAP.

H4a-b: Religiosity (level/dimension) moderates the relationship between DAC (technical skills) and the Muslim/non-Muslim-EAP.

H5a-b: Religiosity (level/dimension) moderates the relationship between DAC (technologies and tools expertise) and the Muslim/non-Muslim-EAP.

## Methods

### Research design and sample

Survey questionnaires ( Underlying data) ( Jaffar et al., 2021) were distributed among final year accounting students at one private university in Malaysia. There were 201 students and all of them participated in the survey. These students had undergone a six-month auditing internship program thus deemed suitable to be used as proxies for EAs. This approach is similar with Ashton and Kramer (1980) who also used students as surrogates for auditors.

DAC was measured by analysing: personal capabilities, professional expertise, technical skills, and technologies and tools expertise as proposed by Strengell’s (2017) ( Underlying data) ( Jaffar et al., 2021). Religiosity (beliefs and practices) was adapted from Mahdzan, Zainudin, Che Hashim and Sulaiman’s (2017). EAP was measured using Asare and Cianci’s (2009) technique, where respondents were given a hypothetical inventory audit case and asked to indicate the likelihood of recommending the inventory to be written off. A pilot test with 50 students revealed that the questionnaire needed no changes. Finally, the questionnaires were sent to the actual respondents.

### Models of the study

Models of this study were as follows:

Model 1:

Perf=β0+β1Personal+β2Professional+β3Technical+β4Tools+e

Model 2:

Perf=β0+β1Personal+β2Professional+β3Technical+β4Tools+β5Personal∗ReliLevel+β6Professional∗ReliLevel+β7Technical∗ReliLevel+β8Tools∗ReliLevel+e

Model 3:

Perf=β0+β1Personal+β2Professional+β3Technical+β4Tools+β5Personal∗Faith+β6Professional∗Faith+β7Technical∗Faith+β8Tools∗Faith+β9Personal∗Virtue+β10Professional∗Virtue+β11Technical∗Virtue+β12Tools∗Virtue+β13Personal∗Optional+β14Professional∗Optional+β15Technical∗Optional+β16Tools∗Optional+e

Model 1 depicts the relationship between DAC and EAP. Level of religiosity (Model 2) and religiosity dimensions (Model 3) were included to test the moderating effects of religiosity on the relationship between DAC and EAP.

## Results and statistical analysis

This study used SPSS Version 26 to analyse the data. Specifically, linear regression was applied to test all the three models of the study. The linear regression was performed separately for Muslim and non-Muslim EAs. Exploratory factor analysis (EFA) was performed to identify the latent constructs of religiosity, and means analysis was used to determine the level of religiosity. Details of the data analysis were presented in the following sections.

### DAC and performance

Linear regression was performed separately for Muslim and non-Muslim EAs. For Muslim EAs, the result exhibited a close to moderate level of R square (42.2%) which signified that variation of the Muslim-EAP was almost moderately explained by their DAC. DAC statistically (p=0.000) predicted the Muslim-EAP. Coefficient results showed that DAC (personal capabilities) was the main predictor (p = 0.000) of the Muslim-EAP. Results of Model 1 for non-Muslim EAs revealed a weak level of R square (1.9%) denoting that the variation of the non-Muslim-EAP was weakly explained by their DAC. ANOVA displayed p = 0.536 indicating that DAC did not statistically predict the non-Muslim-EAP.

### Religiosity

Using factor loading of 0.6, EFA was performed to identify the latent constructs of religiosity ( Hair, Sarstedt, Hopkins & Kuppelwieser, 2014). Only 21 items were used to represent the latent constructs of religiosity for Muslim EAs. The names of the factors were the same as those used by Mahdzan et al. (2017) who adopted Wan Ahmad, Ab. Rahman, Ali and Che Seman’s (2008) and Tiliouine and Belgoumidi’s (2009) measurement scale. Factor one, two and three demonstrated high reliability scores with Cronbach’s alpha between 0.843 and 0.971. This study used 19 items to capture the non-Muslim EAs’ religiosity constructs. The same procedures were applied to the religiosity data of the non-Muslim EAs. Based on the EFA results, five items were discarded because of low factor loadings. The remaining 14 items produced a five-factor solution. Following the test, factor one produced a Cronbach’s alpha value of above 0.8 which was considered good. The Cronbach’s alpha values for factors two and three were 0.752 and 0.629, respectively, which were considered acceptable. Finally, only 12 items were used to represent the latent constructs of religiosity for the non-Muslim EAs.

### Level of religiosity

Muslim religiosity level was measured by the religiosity strength represented by Casual, Moderate and Devout. Similar to Mahdzan et al. (2017), a grand mean was computed as follows:

• Casual - mean scores (μ) were 0.5; standard deviation of 2.915 (μ ≤ 2.915)
• Moderate - mean scores were between 2.925 and 3.415 (2.915 < μ > 3.415)
• Devout - mean scores above 3.415 (≥ 3.415)

Using similar procedures, the non-Muslim EAs’ religiosity strength was computed as follows:

• Casual - mean scores were 0.5; standard deviation of 2.411 (μ ≤ 2.411)
• Moderate - mean scores were between 2.411 and 2.911 (2.411 < μ > 2.911)
• Devout - mean scores above 2.911 (≥ 2.911)

### Moderating role of level of religiosity

In Model 2, linear regression on DAC, level of religiosity and Muslim-EAP exhibited moderate R square (65%) signifying that variation of the Muslim-EAP was moderately explained by DAC and level of religiosity. The ANOVA results displayed p = 0.000 indicating that the Muslim-EAP was significantly predicted. The subsequent results showed that level of religiosity has significant moderating effect (p = 0.054) on the relationship between DAC (technologies and tools expertise) and the Muslim-EAP. The technologies and tools expertise, however, has statistically significant negative main effect on the Muslim-EAP at p = 0.030. This result denoted that the higher the DAC (technologies and tools expertise), the lower the Muslim-EAP. The inclusion of the level of religiosity has improved the overall model fit as evidenced by the higher R square (65%) compared to Model 1’s R square (49%).

Regression results for DAC, level of religiosity and non-Muslim-EAP indicated low R square (4.2%) demonstrating that variation of the non-Muslim-EAP was weakly explained by the DAC and level of religiosity. ANOVA results showed p = 0.556 indicating that the non-Muslim-EAP was not statistically predicted. The inclusion of the level of religiosity slightly improved the overall model fit as evidenced by the higher R square (4.2%) compared to Model 1’s R square (1.9%). Nonetheless, the R square is still considered as low.

### Dimensions of religiosity

Wan Ahmad et al. (2008) introduced four dimensions of Islamic religiosity: Faith, Virtue, Obligation, and Optional. Moderating effects of religiosity dimensions on the relationship between DAC and the EAP were predicted in Model 3 using linear regression analysis.

Regression results showed only the Virtue dimension has significant effect on the relationship between DAC and the Muslim-EAP but not Faith and Optional dimensions. R square = 61.9% suggested that variation of the Muslim-EAP was moderately explained by DAC and Virtue, as supported by ANOVA results of p = 0.001. Subsequent results showed that DAC (personal capabilities) has statistically significant positive main effects on Muslim-EAP at p = 0.011, while DAC (technologies and tools expertise) has statistically significant negative main effects on the Muslim-EAP at p = 0.10 (p < 0.1). The result also demonstrated that Virtue as a dimension of religiosity has significant moderating effect (p = 0.098, using significant level of p < 0.1) on the relationship between DAC (personal capabilities) and the Muslim-EAP. However, the coefficient is -2.084 which denoted the negative direction of the moderating effect of Virtue. In other words, the higher the religiosity (Virtue) the more negative the effect of DAC (personal capabilities) on the Muslim-EAP. Nonetheless, it can be concluded that Virtue as a dimension of religiosity has improved the overall model fit as evidenced by the higher R square (61.9%) compared to Model 1’s R square (49%).

For non-Muslim EAs, the regression results showed only Faith dimension has significant effect on the relationship between DAC and the non-Muslim-EAP but not Virtue and Optional dimensions. The regression result for Faith showed a moderate R square (8.5%) suggesting that variation of the non-Muslim-EAP was weakly explained by DAC and Faith as a dimension of religiosity. The ANOVA results displayed p = 0.076 (p < 0.1), signifying that the non-Muslim-EAP was significantly predicted. The subsequent results showed that DAC (technologies and tools expertise) has statistically significant positive main effects on the non-Muslim-EAP at p = 0.007. Faith as a dimension of religiosity has a significant moderating effect (p = 0.002) on the relationship between DAC (technologies and tools expertise) and the non-Muslim-EAP. However, the coefficient is −0.624 which denoted the negative direction of the moderating impact of Faith. In other words, the higher the religiosity (Faith) the more negative the effect of DAC (technologies and tools expertise) on the non-Muslim-EAP. Nevertheless, the inclusion of Faith as a dimension of religiosity has improved the overall model fit as shown by the higher R square (8.5%) compared to Model 1’s R square (1.9%). Nonetheless, the R square is still considered as very low.

## Discussion

In summary, H1a is accepted for Muslim EAs but not for the non-Muslim EAs. This finding indicates that DAC (personal capabilities) improves the Muslim-EAP. This suggests that personal qualities such as creativity and stress tolerance can improve Muslim EAs performance. This finding corroborates with CPT and Ebrahimi and Hassanein (2018) who demonstrated that DAC has a significant positive relationship with firm’s decision-making performance. As for non-Muslim EAs, H1d is accepted (under Model 3). This finding demonstrates that DAC (technologies and tools expertise) enhances the non-Muslim EAs performance. Obviously, expertise in data analytics technology and methods can enhance the performance of the non-Muslim EAs. This finding is consistent with CPT and Ghasemaghaei, Ebrahimi and Hassanein (2018) regarding the potential impact of DAC on performance. Although the finding for H1d for the Muslim EAs is significant, the relationship is negative (under Model 2). The finding suggests that the higher the skill in data analytics technologies and tools, the lower the performance of the Muslim EAs. This could be due to the nature of EAs' work which relies generally on professional opinion. In addition, Omitogun and Al-Adeem (2019) found that EAs lack relevant technical skills and were unfamiliar with related data analysis tools, except Excel. Both H1b and H1c are not accepted. These findings demonstrate that DAC (professional expertise and technical skills) does not influence the EAP. Professional expertise requirements such as problem-solving skills and reporting are among expertise already required by the profession. This finding corroborates Weber’s (2020) revelation that the EAs were not well prepared for data analytics and that they only had a moderate sense of urgency to become competent in analytics, owing to their employers’ lack of demand for analytics.

H2a, H3a, and H4a are not accepted, implying that level of religiosity has no influence on the relationship between DAC (personal capabilities, professional expertise, and technical skills) and the EAP which is inconsistent with prior research by Ali et al. (2015). The insignificant results may be due to the fact that in the audit profession, the EAs are required to comply with the Professional Code of Ethics. For Muslim EAs, H5a is accepted, but not for non-Muslim EAs. This suggests that the relationship between DAC (technologies and tools expertise) and the Muslim-EAP is moderated by level of religiosity. This finding also shows that the more religious the Muslim EAs are – which indicates that they are honest, ethical or trustworthy – the greater the favourable influence of DAC (technologies and tools expertise) on the Muslim-EAP.

H3b and H4b are not accepted, denoting that religiosity (Faith, Virtue and Optional) does not moderate the relationship between DAC (professional expertise and technical skills) and the EAP. These findings do not corroborate Ali et al. (2015) and this may be due to the fact that the EAs already conform to the Code of Professional Ethics. In the case of H2b, however, religiosity (Virtue) significantly moderated the relationship between DAC (personal capabilities) and the Muslim-EAP, but not for non-Muslim EAs. Nonetheless, the direction is not consistent with the prediction of this study that the more religious Muslim EAs are, the lesser the impact of DAC (personal capabilities) on their performance. Likewise, for H5b, religiosity (Faith) significantly moderated the relationship between DAC (technologies and tools expertise) and the non-Muslim-EAP but not for Muslim-EAP. Nevertheless, the direction is not consistent with the prediction of this study that the more religious non-Muslim EAs are, the lesser the impact of DAC (technologies and tools expertise) on their performance.

This study acknowledged one limitation of this study that was the use of students as proxy for external auditors which could cause problems with generalizability. Nevertheless, because these students had undergone a six-month internship at the accounting firms, this constraint was minimised. As a result of this exposure, they were qualified to act as external auditors' surrogates.

## Conclusion

In conclusion, this study reveals that having DAC is critical for EAs because it can improve their performance. This requires the EAs to acquire data analytics skills for rendering audit services. Furthermore, accounting firms should make data analytics training a priority for their staff. Additionally, religiosity, which has been inadequately tested in DAC literature, provides insights into the significant role of religiosity in EAP. Future research could investigate the cost and benefits of using data analytics in enhancing audit efficiency.

## Ethical approval

This research received an ethical approval from the Research Ethics Committee (REC) of Multimedia University (Approval Number EA2302021).

## Data availability

### Underlying data

Figshare: Data Analytics Competency and Religiosity Influence on External Auditors’ Performance in Malaysia

https://doi.org/10.6084/m9.figshare.16803472 ( Jaffar et al. 2021)

This project contains the following underlying data:

Data (1): The dataset comprises of demographic data of the external auditors, data analytics competency items, religiosity items and performance.

Data file (2): Questionnaire used to collect data on the external auditors’ demographic profile, data analytics competency, religiosity and performance.

Data are available under the terms of the Creative Commons Attribution 4.0 International license (CC-BY 4.0)

## Author contributions

Nahariah Jaffar, Abdul Aziz Ahmad and Noor Adwa Sulaiman were involved in conceptual framework, methodology, analysis, discussion, and writing of the article.
