# Supplementary material for: Data analytics competency and religiosity influence on external auditors’ performance in Malaysia
Source: F1000Res. 2022 Feb 14;10:1133. Originally published 2021 Nov 9. [Version 2] doi: 10.12688/f1000research.73206.2 (PMC8847705; doi:10.12688/f1000research.73206.2)
Supplement: Peer review history

# Peer review: Reviewer response for version 2

**DOI:** 10.5256/f1000research.121243.r123532

**Published:** 2022-02-15

**Authors:** Prem Lal Joshi

**Affiliations:** 1 Western Regional Centre, ICSSR, Mumbai, Maharashtra, India

I have read carefully the replies to my comments by the authors. I am satisfied with the authors' replies. Approved.

Is the work clearly and accurately presented and does it cite the current literature?

Yes

If applicable, is the statistical analysis and its interpretation appropriate?

Yes

Are all the source data underlying the results available to ensure full reproducibility?

Yes

Is the study design appropriate and is the work technically sound?

Yes

Are the conclusions drawn adequately supported by the results?

Yes

Are sufficient details of methods and analysis provided to allow replication by others?

Yes

Reviewer Expertise:

NA

I confirm that I have read this submission and believe that I have an appropriate level of expertise to confirm that it is of an acceptable scientific standard.

# Peer review: Reviewer response for version 1

**DOI:** 10.5256/f1000research.76844.r99721

**Published:** 2022-01-24

**Authors:** Amal Hayati Ahmad Khair

**Affiliations:** 1 Leicester Castle Business School, De Montfort University, Leicester, UK

***My comments:***

 This is a potentially interesting paper. The author/s have an interesting context and study involving data analytics competency and religiosity, in which the former is developed based on four dimensions of competency (i.e. personal capabilities, professional expertise, technical skills, and technology and tools expertise) and the latter, as the moderating factor, is developed based on two measurements of religiosity (i.e. level and dimension), in which their impact were tested on external auditors’ performance in Malaysia.

 It addresses an important topic, especially since some have proposed that religiosity positively influences individual performance ^1^ and organisational behaviour and thus has substantial economic implications. ^2^ Similarly, some have argued that competency has significant impact on performance. ^3^ However, as currently written, the paper seems to be at a very early stage of development and thus considerable development is needed. Despite the rather small temporal and spatial extent of the study on external auditors’ performance (EAP), it should make an important contribution of behavioural studies especially in the auditing within Islamic context.

 The paper has initiated discussions on the issues associated with competency and religiosity on the EAP. However, it exhibits a lack of organisation and coherent arguments in some instances. For example, starting from page 3 onwards, some of the ideas and arguments have not been developed adequately, especially with the hypotheses’ development. Although the paper has attempted to develop the hypotheses with reference to the literature, the arguments were inadequately discussed and have not been linked properly in some instances. For example, under the ‘Dimensions of Religiosity’ section on page 5, four dimensions have been used for Islamic religiosity (i.e. faith, virtue, obligation, optional), but these clash with the abstract, which included only three dimensions (i.e. faith, virtue and optional). Another example, the two measurements of religiosity (i.e. level and dimension), which were introduced in ‘Competence performance theory and hypotheses’ section on page 3, could have been explained clearly how these two dimensions were utilised as moderating factors in the study. Some parts of the methodology sections could have been explained better, for example what it is meant by casual, moderate and devout (refer page 4) for religiosity strength and its link to the EAP.

 In term of argument and methodology, the paper is built on appropriate known concepts, and research is properly designed with correct methodology. However, the discussion on research design and sample could have explained the distribution between Muslim and non-Muslim external auditors. The results could have been presented in tables so that they could be understood and communicated better. Nevertheless, the results have been analysed appropriately and conclusions have been adequately tied together the other elements of the paper in term of the research objective, literature reviews and results.

 In general, the paper clearly expresses its case by taking into considerations all aspects in term of expected knowledge of readers and appropriate language used. However, it contains a number of typographical errors, occasional stylistic or grammatical flaws, corrections to references, etc. Further proof reading would benefit the audience.

**Comments to the Author/s:**

 The subject of your paper is important and the empirical material you have collected holds potential. However, I believe the paper needs considerable development, particularly around the clarity of the concepts mobilised and model development and cannot be given the status approved until these revisions are made. The highlighted comments offer a number of important observations and suggestions for further improvement.

Is the work clearly and accurately presented and does it cite the current literature?

Yes

If applicable, is the statistical analysis and its interpretation appropriate?

Yes

Are all the source data underlying the results available to ensure full reproducibility?

Partly

Is the study design appropriate and is the work technically sound?

Yes

Are the conclusions drawn adequately supported by the results?

Yes

Are sufficient details of methods and analysis provided to allow replication by others?

Partly

Reviewer Expertise:

My primary research interests are accounting fraud/bribery/corruption, fraudulent accounting practices, auditing, ethics, and corporate governance.

I confirm that I have read this submission and believe that I have an appropriate level of expertise to confirm that it is of an acceptable scientific standard, however I have significant reservations, as outlined above.

## References

[1] : THE INFLUENCE OF EMOTIONAL INTELLIGENCE AND ORGANIZATIONAL CITIZENSHIP BEHAVIOR TOWARDS OFFICER PERFORMANCE WITH THE ORGANIZATION AND RELIGIOSITY AS A MODERATING VARIABLE (STUDY ON THE EMPLOYEES OF THE EDUCATION AND CULTURE DEPARTMENT IN DEMAK). *International Journal of Islamic Business Ethics* .2018;3(1) : 10.30659/ijibe.3.1.423-436 10.30659/ijibe.3.1.423-436

[2] : Local religiosity, workplace safety, and firm value. *Journal of Corporate Finance* .2021;70: 10.1016/j.jcorpfin.2021.102093 10.1016/j.jcorpfin.2021.102093

[3] : Motivation as An Intervening Variables Influence Competency on the Performance of Cooperative Managers. *Journal of Management and Entrepreneurship Research* .2021;1(2) : 10.34001/jmer.2020.12.01.2-11 10.34001/jmer.2020.12.01.2-11

# Response

**Published:** 2022-02-11

**Authors:** nahariah jaffar

Dear Prof.

 Thank you for reviewing our manuscript. We are grateful for the insightful comments you have shared.

 The following are our responses to your comments:

 1- Lack of organisation and coherent arguments in hypotheses development.

 Our response: A revision was made to improve the clarity of the hypotheses development section. Additional literature was added to explain the moderating role of religiosity.

 2- Unclear on Dimensions of Religiosity.

 Clearer explanation was made in the religiosity section regarding the adoption of religiosity dimensions in the study that was based on high factor loading.

   

 3- Unclear on Level of Religiosity.

 Clearer explanation was provided in the Level of Religiosity section regarding the meaning of the three levels of religiosity (casual, moderate, devout).

 We hope that these responses are acceptable and look forward to your feedback. Thank you again for your time in reviewing our manuscript.

# Peer review: Reviewer response for version 1

**DOI:** 10.5256/f1000research.76844.r99723

**Published:** 2022-01-18

**Authors:** Khalid Al-Adeem

**Affiliations:** 1 College of Business Administration, King Saud University, Riyadh, Saudi Arabia

- The hypotheses are not deduced from a theory or at least literature. For the hypothetico-deduction to be properly implemented, hypotheses need to be deduced from a theory/theories or at least prior literature on the phenomenon being investigated. Without doing so, the work is merely speculative
- Furthermore, the linear regression does not count for the error term in models. However, I see that the authors included the error term in the three models. The author could have employed Structural Equation Modeling (SEM) to count for the error term, and test the three models once as opposed to test them separately.
- With regard to identifying the “latent constructs of religiosity”, I suggest further performing Confirmatory Factor Analysis (CFA) to confirm the loading and assure the existence of such a contract. That is, to validate what the Exploratory Factor Analysis (EFA) suggests.
- There are grammatical errors in this paper, it should be gone over again and these should be corrected.
- The authors write, 'Meanwhile, religiosity (faith) has significant moderating effect on the relationship between data analytics competency (technologies and tools expertise) and non-Muslim external auditors’ performance',
  I suggest writing the statement this way: 
- *“Meanwhile, religiosity (faith) has a significant moderating effect on the relationship between data analytics competency (technologies and tools expertise) and non-Muslim external auditors’ performance”.*

Is the work clearly and accurately presented and does it cite the current literature?

Yes

If applicable, is the statistical analysis and its interpretation appropriate?

No

Are all the source data underlying the results available to ensure full reproducibility?

Yes

Is the study design appropriate and is the work technically sound?

No

Are the conclusions drawn adequately supported by the results?

Partly

Are sufficient details of methods and analysis provided to allow replication by others?

Yes

Reviewer Expertise:

Development of Accounting Thought; IFRS; Accounting Research Methodology; Epistemology; Auditor Independence;

I confirm that I have read this submission and believe that I have an appropriate level of expertise to confirm that it is of an acceptable scientific standard, however I have significant reservations, as outlined above.

# Response

**Published:** 2022-02-11

**Authors:** nahariah jaffar

Dear Prof

 Thank you for reviewing our manuscript. We are grateful for the insightful comments you have shared.

 The following are our responses to your comments:

 1- Hypotheses not deduced from theory

 Our response: A revision was made to improve the clarity of the hypotheses development section. This study adopted Competence Performance Theory (CPT) by Korossy (1999) to predict the effects of DAC on the EAP. In addition, additional literature were added to explain the moderating role of religiosity.

 2- Adoption of SEM

 Our response: The revised manuscript retained the existing data analysis because the current software used in the study was able to analyse the current data and achieved the objectives of the study.

 3- Grammatical error

 Our response: the manuscript has been sent for English proof reading.

 4- To improve one statement in the abstract.

 Our response: We have made the necessary changes.

 We hope that these responses are acceptable and look forward to your feedback. Thank you again for your time in reviewing our manuscript.

# Peer review: Reviewer response for version 1

**DOI:** 10.5256/f1000research.76844.r99722

**Published:** 2021-12-13

**Authors:** Prem Lal Joshi

**Affiliations:** 1 Western Regional Centre, ICSSR, Mumbai, Maharashtra, India

The issues studied in this research are contemporary in auditing research, and limited work has been undertaken so far in this area. Therefore, any research on the effects of data analytics competency on the external auditors’ performance is very welcome. The theme and research questions investigated in this study are highly relevant. Additionally, it is interesting to know the moderating effects of religiosity on data analytics competency and external auditors’ performance relationship.

 This research uses a survey method which is acceptable. The sample size is quite large. The data analytics competency dimensions are indicated by personal capabilities, professional expertise, technical skills, technologies and tools expertise, which may be considered as relevant measures. The religiosity variable is measured by level and dimension (faith, virtue and optional).

 The theory applied in this research is Competence Performance Theory (CPT) as laid out by Korossy (1999), which is also relevant to this type of research. ^1^ The hypotheses formulated are duly supported by prior research.

 The study findings state that data analytics competency (personal capabilities) has a positive significant effect on the Muslim external auditors’ performance. However, data analytics competency does not affect the performance of non-Muslim external auditors. Level of religiosity has a significant moderating effect on the relationship between data analytics competency (technologies and tools expertise) and Muslim external auditors’ performance. However, the level of religiosity has no moderating effect on the relationship between data analytics competency and the performance of non-Muslim external auditors.

 My main concern is that the authors have not provided clear reasons or justifications for these differences in findings between Muslim external auditors and non-Muslim external auditors. Do both the groups acquire different education training and skills with respect to DAC in Malaysia? Why does this difference occur? Can these findings be generalized to other environments?

 I recommend the manuscript for approval subject to addressing the above minor comments.

Is the work clearly and accurately presented and does it cite the current literature?

Yes

If applicable, is the statistical analysis and its interpretation appropriate?

Yes

Are all the source data underlying the results available to ensure full reproducibility?

Yes

Is the study design appropriate and is the work technically sound?

Yes

Are the conclusions drawn adequately supported by the results?

Yes

Are sufficient details of methods and analysis provided to allow replication by others?

Yes

Reviewer Expertise:

NA

I confirm that I have read this submission and believe that I have an appropriate level of expertise to confirm that it is of an acceptable scientific standard, however I have significant reservations, as outlined above.

## References

[1] : Modelling knowledge as competence and performance. *Psychology Press* .1999;

# Response

**Published:** 2022-02-11

**Authors:** nahariah jaffar

Dear Prof

 Thank you for your time in reviewing our manuscript. We are greatly appreciative of the insightful comments that you have provided. 

 Our responses to your comments are as follows:

 1- Justification for the differences in findings between Muslim external auditors and non-Muslim external auditors.

 Our response: The revised version provided the justification (in the discussion section) that one possible reason might be due to the level of support provided by the auditing firms. Hence, further research is needed to understand other possible factors affecting the DAC and subsequently the EAP.

 2- Generalizability of the findings to other environments.

 Our responses: In the discussion section of the revised version, it is stated that the study provided an initial investigation into the effects of religiosity on EAP. Future research might want to incorporate other contributing factors affecting EAP such as organizational culture to understand the effects of various factors affecting performance in other environment or setting.

 We hope that our responses are acceptable and look forward to your feedback. Thank you again for your time in reviewing this manuscript.
